# A New Mode of Counter Extension

**Published:** 1861-12

**Authors:** E. Andrews

**Affiliations:** Professor of Surgery in Lind University, etc., etc.


ARTICLE IV.

### A NEW MODE OF COUNTER-EXTENSION.

By E. ANDREWS, A. M., M. D.,

PROFESSOR OF SURGERY IN LIND UNIVERSITY, ETC., ETC.

Every device which renders the treatment of fractures less irksome to the patient, is a blessing to the surgeon; hence the publication of the following plan for making counter-extension, which is, so far as I know, mostly new; but as it is very simple, possibly it may have already been practiced by others, who have not taken the trouble to publish it. If so, I cheerfully waive all claim to priority.

Every surgeon who has tried the modern plan of extension in fractures of the femur, by means of adhesive straps, has been struck with its efficiency and the perfect freedom which it ensures the patient from all pain and soreness about the leg and foot. As compared with the old fashioned gaiter, which, after a few days' use, hurts the instep and the heel severely, it is a surprising improvement. This complete immunity from suffering in extension by straps, has often prompted efforts to produce counter-extension, also, by similar means, and thus avoid the pain which always results from the perineal band. Indeed, this is a very important point, for in almost all cases of shortening after fractures of the femur, it will be found that there was no physical impossibility of keeping up full extension, but that the sufferings of the patient from the extending and counter-extending apparatus, either caused him to rebel and loosen his dressings without permission, or else wrought upon the compassion of the surgeon so far, as to induce him to do the same thing. A mode of counter-extension, therefore, which is as painless as the adhesive-strap extension, is equivalent to the power of ensuring unions of the fractured bones, at their full original length.

Efforts, in this direction, have been made by the use of oblique straps, passing across the body from the top of a Dessault's splint, but in my hands I have found them failures.

The traction is too nearly transverse, and presses the splint too strongly against the hip. To avoid this difficulty, I have made use of the following device: I first cut three adhesive straps, each a yard and a half long, and two and a half inches wide; one of these being warmed, is applied to the back, on the same side as the fracture, extending from the waist to the top of the shoulder, and then down the breast, on the same side, as far as the waist in front. At the top of the shoulder it is left loose for the attachment of a hook. The second strap extends from the top of the same shoulder, obliquely down the back and breast, towards the opposite side, until the ends cross each other on the side of the waist. The third strap is placed as a belt around the body, to confine the ends of the others. Next, I take a Dessault's, or any other long splint to which I have previously had attached, at its superior extremity, a bent iron, which, passing over the curve of the shoulder, is hooked into the loose part of the two adhesive-straps where they pass over the top of the shoulder. The lower extremity of the splint is attached to the leg and foot, as usual, by adhesive-strap extension.

My experience with this dressing, is very happy. The traction is distributed over so large a surface, that it occasions no pain, but allows the surgeon to apply, with impunity, any amount of force necessary to make complete extension. A very cheap and efficient form of this apparatus may be constructed by attaching a curved iron, by rivets, to the top of a common Dessault's splint. A more elegant one may be made, by having half the length of the splint made of a brass tube, and the other half, of a rod with a screw surface to slide into it. On the rod, turns a nut, which, resting against the end of the tube, regulates the length, and makes extension. To the lower end, is attached the cross-piece for the foot, and to the upper extremity, the curved shoulder-piece. The whole is strong, simple, and elegant, and not costly. A variety of other forms will readily suggest themselves to any ingenious man.

I think a little experience will satisfy any one that, in fractures of the thigh, counter-extension from the top of the shoulder, by means of adhesive straps, is by far the best way to fulfill a very troublesome and difficult indication.

